# Relationship of Methylenetetrahydrofolate Reductase (MTHFR) C677T Variation With Susceptibility of Patients With Ischemic Stroke: A Meta-Analysis

**DOI:** 10.7759/cureus.28218

**Published:** 2022-08-20

**Authors:** Pramod Kumar, Aparna Mishra, Manoj K Prasad, Vivek Verma, Amit Kumar

**Affiliations:** 1 Biochemistry, Rajendra Institute of Medical Sciences, Ranchi, IND; 2 Neuroanaesthesiology, All India Institute of Medical Sciences, New Delhi, IND; 3 Medicine, Rajendra Institute of Medical Sciences, Ranchi, IND; 4 Statistics, Assam University, Silchar, IND; 5 Laboratory Medicine, Rajendra Institute of Medical Sciences, Ranchi, IND

**Keywords:** mthfr, methylenetetrahydrofolate reductase, meta-analysis, ischemic stroke, stroke, gene polymorphism, methylenetetrahydrofolate

## Abstract

Discovery and validation of genetic factors for multifactorial and polygenic disorders like stroke are needed to make progress in precision medicine. Although some traditional risk factors for stroke have been identified, they do not fully explain the pathophysiological mechanism of ischemic stroke. The research of genetic risk factors is becoming increasingly relevant in the understanding of stroke mechanisms and the finding of population-specific therapeutic targets. The methylenetetrahydrofolate reductase (MTHFR) gene is involved in homocysteine metabolism, and a high homocysteine level is a risk factor for stroke. Using a meta-analysis technique, we investigated the link between the MTHFR C677T gene polymorphism and the risk of ischemic stroke.

We used the electronic databases PubMed, Medline, Embase, and Google Scholar to find articles in the Journal of Stroke. If heterogeneity was more than 50%, pooled odds ratios (ORs) with 95% confidence intervals (CIs) were calculated using a random-effects model; otherwise, a fixed-effects model was used.

A total of 67 case-control studies with 17,704 cases and 21,981 controls met our inclusion criteria. The Asian population was represented by 41 studies, whereas the Caucasian population was represented by 26. Under the recessive model, a gene polymorphism at the 677 location of the MTHFR gene is related to an elevated risk of ischemic stroke (OR: 1.29, 95% CI: 1.22-1.37, P < 0.001).

People who have the MTHFR C677T gene polymorphism have a greater risk of stroke than people who do not.

## Introduction and background

Stroke has risen to become the second largest cause of mortality in adults and the third leading cause of disability. Understanding the pathogenesis of stroke necessitates the finding of risk factors [1,2]. Traditional risk factors for ischemic strokes, such as hypertension, diabetes, atrial fibrillation, and smoking, have been extensively researched, although they only account for a minor part of stroke risk [3]. Many previously recognized risk factors for stroke do not fully explain the mechanism of stroke because many stroke victims do not have these risk factors [4]. There was a significant genetic susceptibility to ischemic stroke, according to the evidence from twin and familial aggregation of stroke research. Stroke is a complex disease, according to studies, and it may be caused by shared genetic and environmental variables [5]. It has long been known that a variation in the methylenetetrahydrofolate reductase (MTHFR) gene is linked to the risk of stroke [6].

The 5,10-methylenetetrahydrofolate reductase is an important enzyme that regulates the metabolism of homocysteine (Hcy) levels [7]. MTHFR is an enzyme that helps in the conversion of 5,10-methylenetetrahydrofolate to 5-methylenetetrahydrofolate, which further converts Hcy to methionine [8,9]. The MTHFR gene polymorphism is linked to a reduced conversion of 5,10-methylenetetrahydrofolate to 5-methylenetetrahydrofolate, which is responsible for the accumulation of Hcy in the bloodstream due to a slowed remethylation reaction from Hcy [10]. Therefore, the alteration in the function of the MTHFR pathway leads to an increased risk of cerebrovascular disease by elevating the level of Hcy in the circulation. Previous epidemiological studies have observed that polymorphism in the MTHFR C677T position is associated with a higher risk of stroke [11,12]. MTHFR gene is considered important to understand the genetic risk of stroke indicated by the published reports. The evidence of precise association can be estimated by conducting a meta-analysis to quantify the pooled effect size based on earlier reported studies in the literature with a similar objective [13]. As a result, we conducted the biggest meta-analysis of papers published to date to discover the precise relationship between the C677T polymorphism in the MTHFR gene and ischemic stroke.

## Review

Methodology and literature search

We followed the Preferred Reporting Items for Systematic Reviews and Meta-Analyses (PRISMA) guidelines for reporting meta-analysis findings [14]. We conducted a computerized search of MEDLINE, Google Scholar, PubMed, Stroke journal, Web of Science, and Springer for relevant case-control studies from 1997 to 2020. We also looked through references of published manuscripts, editorials, and systematic reviews. The electronic search terms and keywords for obtaining the relevant articles were “MTHFR” OR “MTHFR Polymorphism” OR “MTHFR TT polymorphism” OR “Homocysteine” OR “ischemic stroke in MTHFR TT gene” OR “MTHFR C677T gene in ischemic stroke” OR “MTHFR in stroke.” We fixed the filter so that results were limited to humans and articles published in the English language.

Inclusion and exclusion criteria

Inclusion criteria included the following: (a) studies that used a case-control study design investigating the relationship between the MTHFR C677T gene and the risk of ischemic stroke; (b) studies including ischemic stroke cases and healthy controls; (c) studies that mentioned the diagnostic criteria for ischemic stroke; (d) studies that reported the genotypic frequencies for both cases and controls; (e) studies with patients aged > 18 years; and (f) studies with enough data for extraction for computing pooled effect size.

Studies were excluded (a) in case genotype frequencies could not be extracted; (b) studies conducted on other subtypes of stroke; (c) cohort studies, cross-sectional studies, and randomized controlled trials; and (d) duplicate publications from the same study with overlapping subjects.

Extraction of data and evaluation of methodological quality

We have used the standardized data collection form to extract the data from the included studies. The following important data were extracted for the present study: first author's name, year of article publication, journal in which the article was published, number of genotypes reported in the cases and controls, mean age of cases and controls, and ethnicity. To avoid duplication of the material, we kept only the most recent article or entire study where the same population was reported in multiple publications. Any disputes between the writers were settled through dialogue. For the purposes of the study, ethnicities were divided into two categories: Asian and Caucasian. We also used a quality rating scale created for genetic association studies to assess the methodological quality. Traditional epidemiologic considerations, as well as genetic issues, were included in this scale [15]. The scores ranged from 0 (worst) to 16 (highest).

Pooled odds ratio (OR) with 95% CI was used to determine the pooled effect size [16]. The I2 statistic was used to determine statistically significant heterogeneity. We used the random effects model in case of heterogeneity of more than 50%, otherwise, the fixed effect model was used. The probable publication bias was diagnosed using funnel plots and Egger's linear regression test. An ethnicity-based stratified analysis (Asian vs. Caucasian) was carried out. We opted for a two-sided test with <0.05 treated as statistically significant.

Results

Previously done meta-analysis studies investigating MTHFR C677T polymorphism and ischemic stroke with OR are shown in Table 1 [12,15-30].

**Table 1:** Pooled ORs of risk from studies investigating methylenetetrahydrofolate reductase (MTHFR) C677T polymorphism and ischemic stroke.

S. No.	Year	Authors	Origin	Sample size, case/control	Total studies	Result (OR, 95% CI)
1	2019	Chang et al. [12]	China	0/0	9 studies	1.41 (1.14-1.75)
2	2015	Kumar et al. [15]	India	6310/8297	38 studies	1.31 (1.19-1.44)
3	2014	Zhang et al. [16]	China	7990/6941	68 studies	1.86 (1.50-2.31)
4	2017	Abhinand et al. [17]	India	12,390/16,274	72 studies	1.319
5	2014	Wu et al. [19]	China	5207/5383	30 studies	1.62 (1.32-1.99)
6	2013	Yadav et al. [20]	India	2529/2881	26 studies	2.50 (0.89-6.97)
7	2002	Wald et al. [21]	London	1217/676	7 studies	1.21 (1.06-1.39)
8	2008	Trabetti [22]	Italy	4375/4856	24 studies	-
9	2005	Cronin et al. [23]	Ireland	6110/8760	32 studies	1.37 (1.15-1.64)
10	2004	Casas et al. [24]	London	3387/4597	22 studies	1.24 (1.08-1.42)
11	2002	Clarke et al. [25]	England	344/300	30 studies	-
12	2000	Moller et al. [26]	Denmark	0/0	21 studies	3.97
13	2008	Xu et al. [27]	China	296/216	13 studies	1.55 (1.26-1.90)
14	2009	Xin et al. [28]	China	2806/7636	26 studies	1.44 (1.14-1.80)
15	2016	Song et al. [29]	China	4564/6701	22 studies	1.37 (1.16-1.61)
16	2013	Li et al. [30]	China	2223/2936	19 studies	1.28 (1.17-1.40)

**Figure 1:**
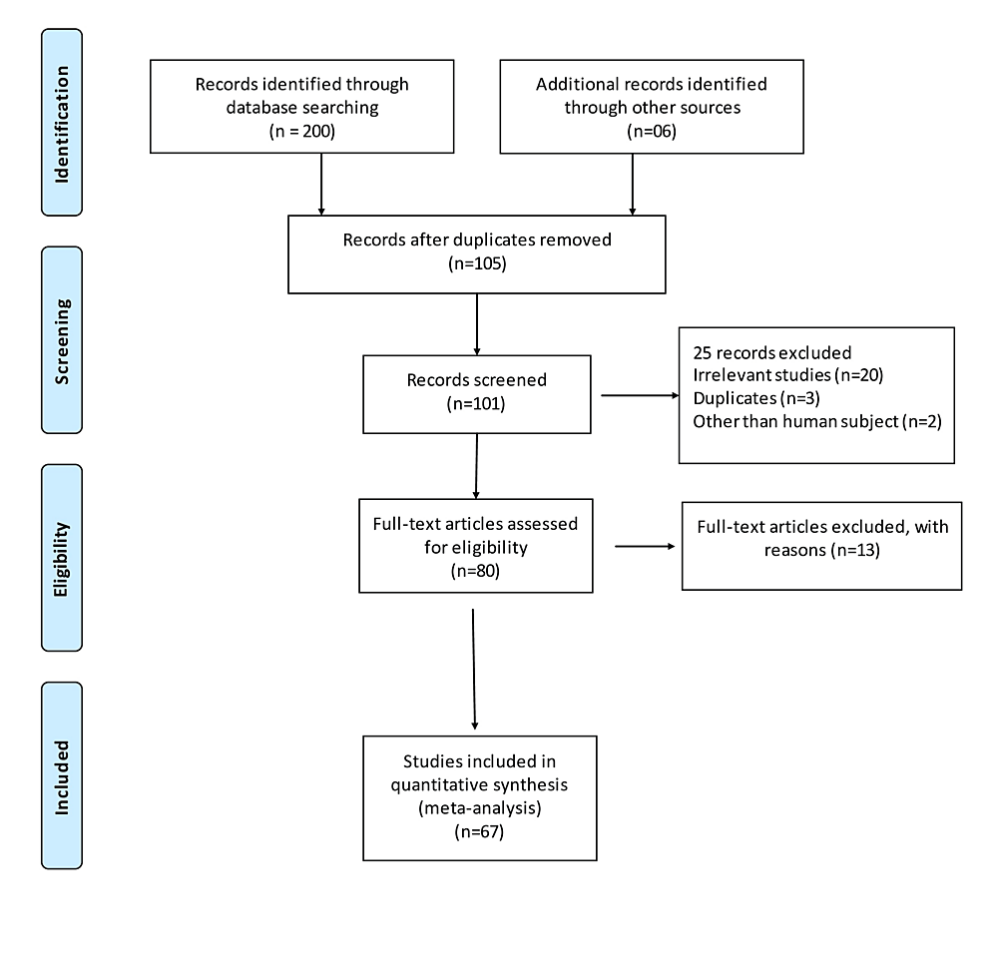
PRISMA flow diagram. PRISMA: Preferred Reporting Items for Systematic Reviews and Meta-Analyses.

**Figure 2:**
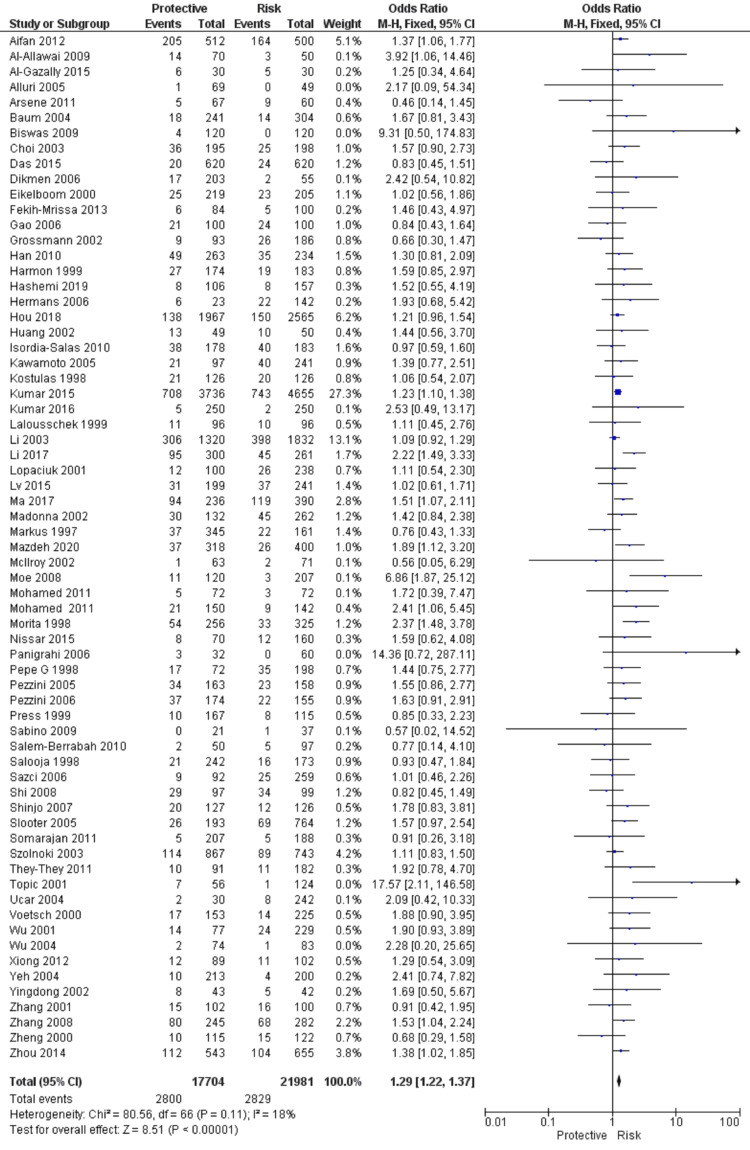
Forest plot and pooled ORs of risk from studies investigating methylenetetrahydrofolate reductase (MTHFR) C677T polymorphism and ischemic stroke. Reference citations: [83], [73], [86], [60], [81], [57], [72], [53], [88], [67], [39], [84], [64], [48], [75], [38], [94], [65], [93], [47], [77], [61], [35], [15], [90], [37], [52], [92], [45], [89], [91], [49], [31], [95], [50], [71], [78], [32], [87], [66], [33], [59], [62], [36], [74], [76], [34], [63], [70], [68], [58], [80], [51], [79], [42], [56], [40], [44], [55], [82], [54], [46], [43], [69], [41], [85].

**Figure 3:**
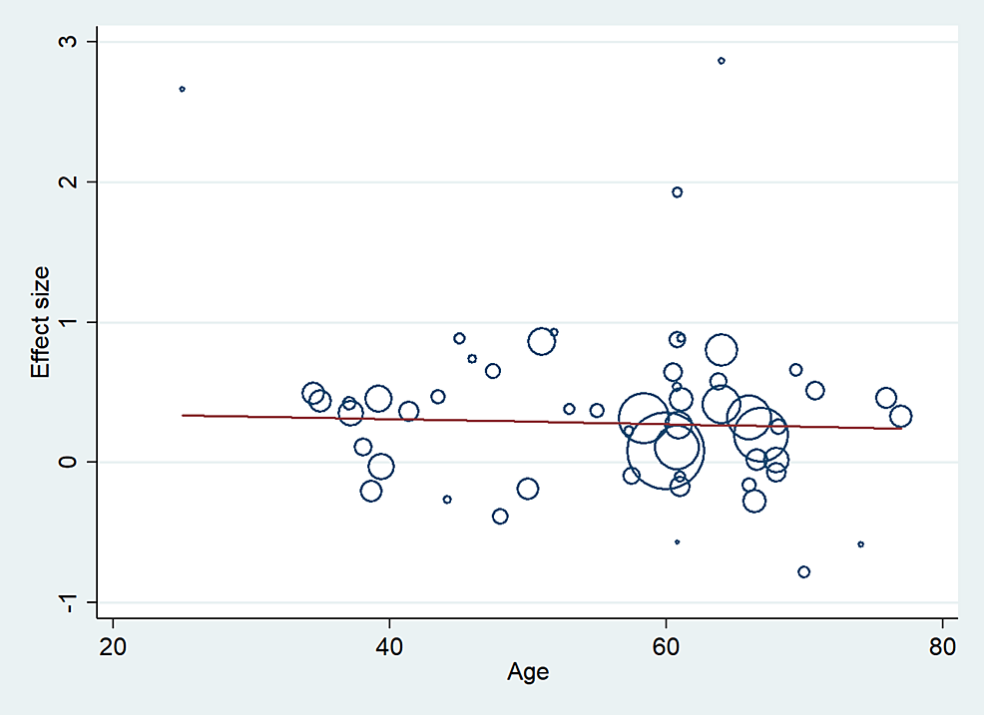
Meta-regression analysis to determine the influence of mean age in the study population with effect size.

**Figure 4:**
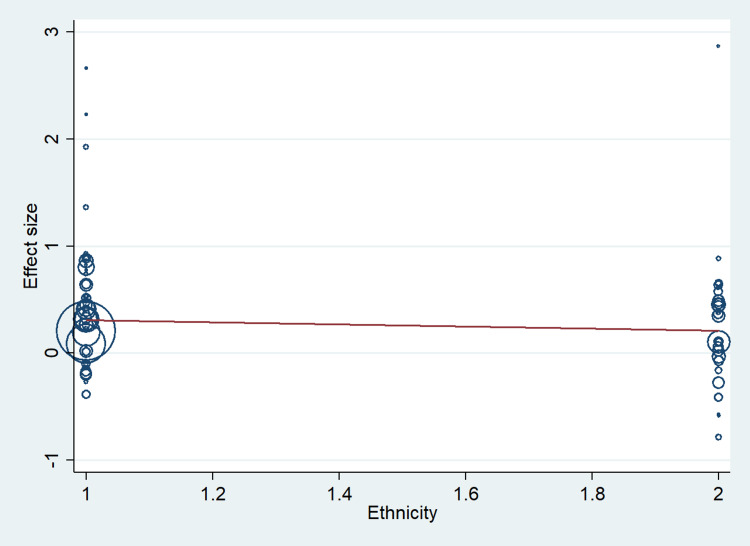
Meta-regression analysis to determine the influence of ethnicity in the study population with effect size.

**Figure 5:**
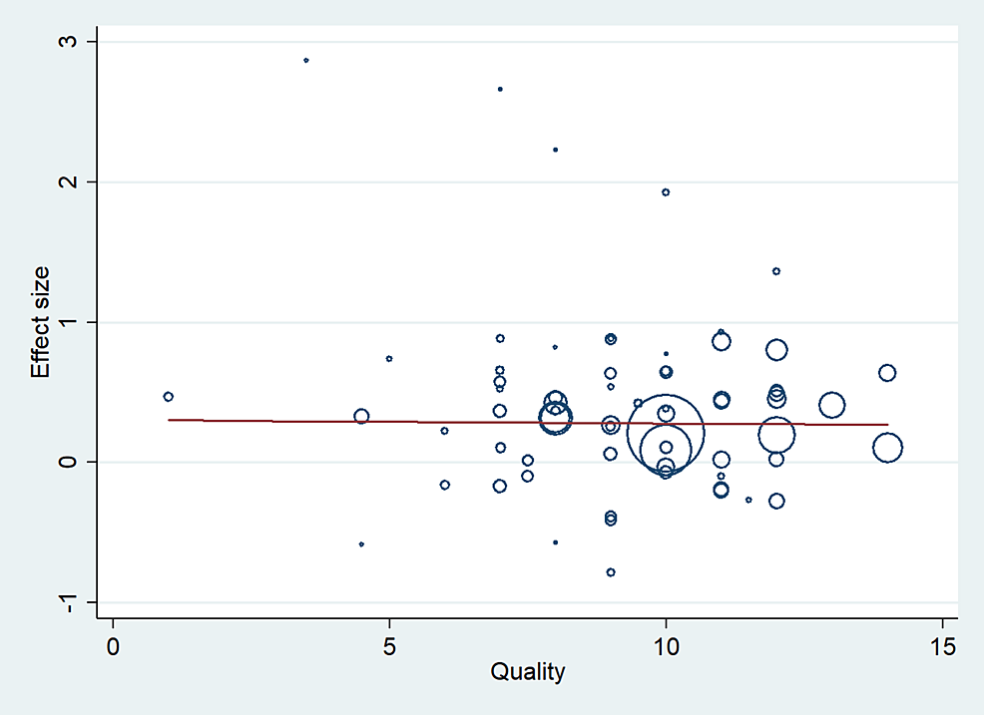
Meta-regression analysis to determine the influence of methodological quality in the study population with effect size.

**Figure 6:**
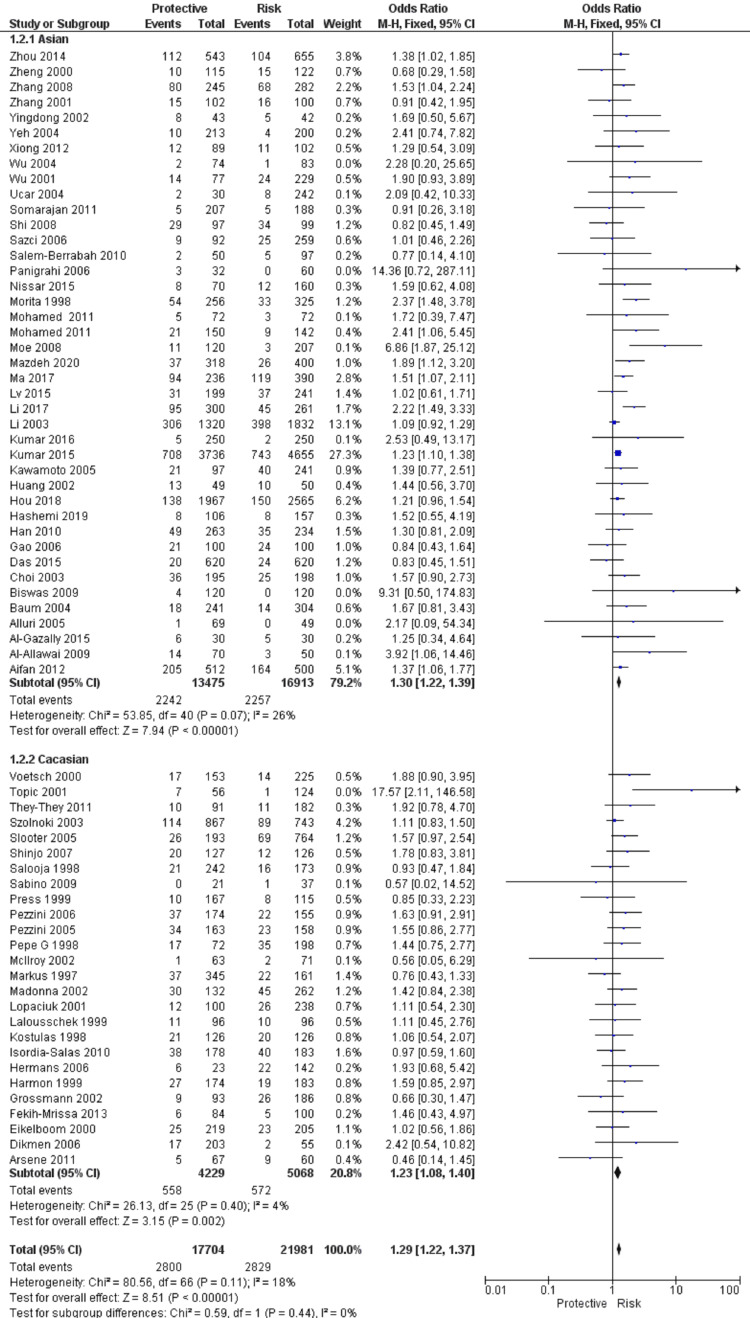
Forest plot and pooled ORs of subgroup (year). Subgroup - Asian studies: [85], [41], [69], [43], [46], [54], [82], [55], [44], [56], [80], [70], [63], [76], [66], [87], [32], [78], [71], [95], [91], [89], [92], [52], [90], [15], [61], [47], [93], [94], [75], [64], [88], [53], [72], [57], [60], [86], [73], [83]. Subgroup - Caucasian studies: [40], [42], [79], [51], [58], [68], [34], [74], [36], [62], [59], [33], [50], [31], [49], [45], [37], [35], [77], [65], [38], [48], [84], [39], [67], [81].

A total of 67 studies that met the inclusion criteria were included in this study, having 17,704 cases and 21,981 controls. The studies were conducted from the period of 1997 to 2020. There were 41 studies from the Asian population and 26 from the Caucasian population. Figure 1 shows the search results. The characteristics of the included studies are presented in Table 2. In this meta-analysis, all studies' genotype data were following the Hardy-Weinberg equilibrium. All included studies’ methodological quality scores ranged from 3.5 to a maximum of 14 (Table 2). MTHFR gene polymorphism at 677 locations is significantly associated with the increased risk of ischemic stroke (OR: 1.29, 95% CI: 1.22-1.37, P < 0.001) (Figure 2). Meta-regression analysis has shown no significant influence on mean age (P = 0.693) (Figure 3), ethnicity (P = 0.71) (Figure 4), and methodological quality in the study population (P = 0.977) with effect size (Figure 5). We stratified the data into two groups based on the results of studies conducted on Asian and Caucasian populations. Subgroup analysis (year-wise) has shown no association in the studies having an OR and corresponding 95% CIs of 1.30 (1.22-1.39) for the Asian population and 1.23 (1.08-1.40) for the Caucasian population (Figure 6).

**Table 2:** Characteristics of studies included in the meta-analysis on the association between MTHFR C677T polymorphism and ischemic stroke. MTHFR: methylenetetrahydrofolate reductase.

S. No.	Year	Study	Origin	Sample size, case/control	Hardy-Weinberg equilibrium (HWE)	Total, male/female	Age	Quality score	
									1	1997	Markus et al. [31]	London	345/161	Yes	287/0	66.4	12	
									2	1998	Morita et al. [32]	Japan	256/325	Yes	0	51	11	
									3	1998	Pepe et al. [33]	Italia	72/198	No	72/198	41.4	7	
4	1998	Salooja et al. [34]	London	242/173	No	68/69	68	10	
5	1998	Kostulas et al. [35]	Sweden	126/126	Yes	0	0	9	
6	1999	Press et al. [36]	Portland	167/115	Yes	126/52	66	6	
7	1999	Lalouschek et al. [37]	Austria	96/96	No	58/38	0	7	
8	1999	Harmon et al. [38]	Ireland	174/183	No	183/174	75.9	8	
9	2000	Eikelboom et al. [39]	Australia	219/205	Yes	195/219	66.6	12	
10	2000	Voetsch et al. [40]	Brazil	153/225	Yes	153/225	0	9	
11	2000	Zheng et al. [41]	China	115/122	Yes	18/12	48	9	
12	2001	Topić et al. [42]	Croatia	56/124	No	92/0	64	3.5	
13	2001	Zhang et al. [43]	China	102/100	Yes	102/100	57.5	7.5	
14	2001	Wu et al. [44]	Japan	77/229	Yes	77/229	60.5	10	
15	2001	Lopaciuk et al. [45]	Poland	100/238	No	51/49	38.1	10	
16	2002	Yingdong et al. [46]	China	43/42	Yes	0	0	7	
17	2002	Huang et al. [47]	China	49/50	Yes	0	55	8	
18	2002	Grossmann et al. [48]	Germany	93/186	No	140/139	0	9	
19	2002	Madonna et al. [49]	Italy	132/262	No	117/145	37.2	10	
20	2002	Mcllroy et al. [50]	Ireland	63/71	No	71	74.1	4.5	
21	2003	Szolnoki et al. [51]	Hungary	867/743	Yes	853/757	60.8	14	
22	2003	Li et al. [52]	China	1320/1832	No	0	60	10	
23	2003	Choi et al. [53]	China	195/198	Yes	195/198	61.1	11	
24	2004	Yeh et al. [54]	China	213/200	No	173/167	45.1	7	
25	2004	Wu et al. [55]	China	74/83	Yes	0	0	8	
26	2004	Uçar et al. [56]	Turkey	30/242	No	201/71	46	5	
27	2004	Baum et al. [57]	China	241/304	Yes	268/0	70.8	12	
28	2005	Slooter et al. [58]	Netherlands	193/764	No	0	39.2	12	
29	2005	Pezzini et al. [59]	Italy	163/158	No	169/0	35	11	
30	2005	Alluri et al. [60]	India	69/49	No	30/10	0	10	
31	2005	Kawamoto et al. [61]	Japan	97/241	Yes	175/0	77	4.5	
32	2006	Pezzini et al. [62]	Italy	174/155	Yes	149/155	34.5	12	
33	2006	Sazci et al. [63]	Turkey	92/259	No	181/168	0	7.5	
34	2006	Gao et al. [64]	China	100/100	Yes	71/71	61	7	
35	2006	Hermans et al. [65]	Belgium	23/142	Yes	23/154	69.4	7	
36	2006	Panigrahi et al. [66]	India	32/60	No	0	25	7	
37	2006	Dikmen et al. [67]	Turkey	203/55	Yes	126/132	61.1	9	
38	2007	Shinjo et al. [68]	Brazil	127/126	Yes	125/0	63.8	7	
39	2008	Zhang et al. [69]	China	245/282	Yes	255/282	0	8	
40	2008	Shi et al. [70]	China	97/99	No	159/37	38.7	11	
41	2008	Moe et al. [71]	Singapore	120/207	Yes	233/94	60.8	10	
42	2009	Biswas et al. [72]	India	120/120	Yes	0	0	8	
43	2009	Al-Allawi et al. [73]	Iraq	70/50	No	64/56	0	12	
44	2009	Sabino et al. [74]	Brazil	21/37	No	24/34	60.8	8	
45	2010	Han et al. [75]	Korea	263/234	Yes	267/234	60.9	9	
46	2010	Salem-Berrabah et al. [76]	Tunisia	50/97	No	53/97	44.2	11.5	
47	2010	Isordia-Salas et al. [77]	Mexico	178/183	Yes	122/120	39.4	10	
48	2011	Mohamed et al. [78]	Malaysia	72/72	Yes	163/129	60.8	9	
49	2011	They-They et al. [79]	Morocco	91/182	Yes	91/182	47.5	10	
50	2011	Somarajan et al. [80]	India	207/188	Yes	0	0	11	
51	2011	Arsene et al. [81]	Romania	67/60	No	53/97	70	9	
52	2011	Mohamed et al. [78]	Malaysia	150/142	Yes	163/129	60.8	9	
53	2012	Xiong et al. [82]	China	89/102	Yes	0/53	68.1	9	
54	2012	Aifan et al. [83]	China	512/500	No	310/202	58.4	8	
55	2013	Fekih-Mrissa et al. [84]	Tunisia	84/100	No	121/63	53	10	
56	2014	Zhou et al. [85]	China	543/655	No	748/452	66	8	
57	2015	Al-Gazally et al. [86]	Iran	30/30	No	90/110	57.3	6	
58	2015	Nissar et al. [87]	India	70/160	Yes	133/97	43.5	1	
59	2015	Kumar et al. [15]	India	6310/8297	Yes	0	0	10	
60	2015	Das et al. [88]	India	620/620	Yes	862/388	50	11	
61	2015	Lv et al. [89]	China	199/241	Yes	245/195	68	11	
62	2016	Kumar et al. [90]	India	250/250	Yes	406/97	51.9	11	
63	2017	Ma et al. [91]	China	236/390	Yes	368/258	64	13	
64	2017	Li et al. [92]	China	300/261	No	257/304	64	12	
65	2018	Hou et al. [93]	China	1967/2565	Yes	2858/0	66.9	12	
66	2019	Hashemi et al. [94]	Southeast Iran	106/157	No	111/154	37.1	9.5	
67	2021	Mazdeh et al. [95]	Iran	318/400	Yes	318/400	0	14	

Publication bias

**Figure 7:**
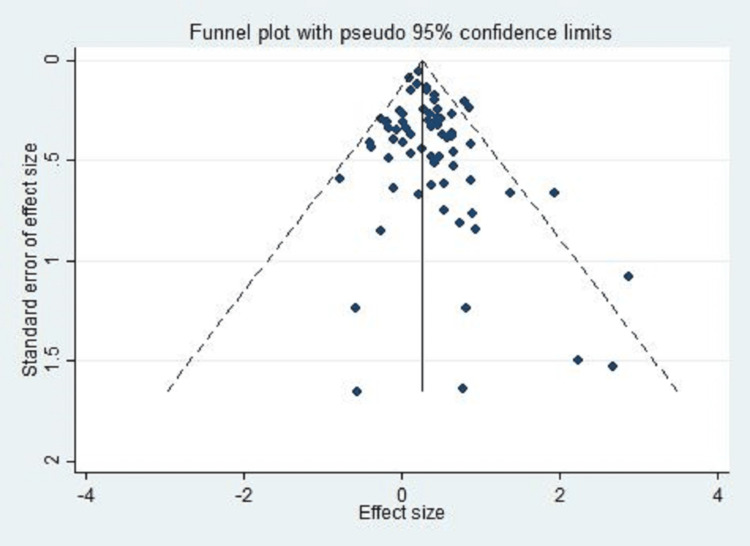
Funnel plot for assessing publication bias.

The probabilities of publication bias arising from the published literature were examined using a funnel plot and the Begg’s and Egger's tests. We observed that there was significant publication bias (P < 0.001), indicating that there were probabilities of publication bias (Figure 7).

Discussion

Our meta-analysis, which included 67 studies, observed that variation at the C677T position of the MTHFR gene might be associated with an increased risk to develop ischemic stroke.

Earlier meta-analyses [16,17] with a substantial number of studies have also shown the significant relationship between C677T variation of the MTHFR gene and increased risk of ischemic stroke (Table 1). However, earlier meta-analyses had limitations to obtain the precise estimate of risk associated with MTHFR gene polymorphism for the risk of ischemic stroke. The meta-analysis published by Zhang et al. [16] recruited studies (68 studies) only from the Chinese population, which limits the generalizability of the study findings. Another meta-analysis reported by Abhinand et al. [17] in 2017 had limitations with the inclusion of the same study multiple times, and inadequate statistical analysis to draw a precise conclusion. This meta-analysis also included studies with cervical artery dissections and venous thrombosis, which would have influenced the pooled effect size to derive a homogenous effect size.

In view of these, our meta-analysis is the largest meta-analysis that used the robust statistical method and methodological quality to derive the precise conclusion regarding the relationship of MTHFR gene polymorphism at 677 positions with the risk of ischemic stroke. In the stratified analysis, the association was found to be higher in the Asian population (OR: 1.30, 95% CI: 1.22-1.39) as compared to the Caucasian population (OR: 1.23, 95% CI: 1.08-1.40). However, in meta-regression analysis, ethnicity did not contribute to the significant heterogeneity in the pooled effect size. These findings indicate that similar type of association between MTHFR gene polymorphism and the risk of ischemic stroke in both Asian and Caucasian populations. Our meta-regression analysis to explore the source of variation in effect size did not observe the significant influence of mean age, methodological quality, and year of publication of articles on the pooled effect size. These observations further strengthen the homogeneous effect of the MTHFR gene polymorphism with an increased risk of ischemic stroke.

MTHFR polymorphism leads to a higher level of Hcy. Hcy is a sulfur-containing amino acid and its remethylation leads to the formation of methionine. In the remethylation process of methionine, the methyl donor for the conversion of Hcy to methionine is done by the reduction of 5,10-methylenetetrahydrofolate to 5-methyltetrahydrofolate by the enzyme MTHFR. Elevated plasma Hcy levels can occur due to defective remethylation of Hcy to methionine because mutations in the MTHFR gene could lead to decreased activity of the MTHFR enzyme [16-18]. Stroke guidelines have included the examination of the Hcy biomarker in young stroke patients as a higher level of Hcy was found to be associated with an increased risk of stroke. It could be effectively treated with vitamin B12 and folic acid supplementation. It has been observed that vitamin supplementation effectively controls the level of Hcy and thereby reduces the risk of stroke [8]. The findings of the present study further strengthen the routine examination of MTHFR gene polymorphism for the prevention of stroke along with Hcy levels.

## Conclusions

This meta-analysis sustains the notion of the association of MTHFR gene polymorphism with an increased risk of ischemic stroke. The observed pooled effect size had insignificant heterogeneity, which further strengthens the findings observed in the current study. The study is limited by the presence of publication bias. The association of MTHFR gene polymorphism was found to be higher in the Asian population compared to Caucasians. MTHFR gene polymorphism screening may be included in the guidelines for the prevention and screening of subjects with higher susceptibility to stroke.

## References

[1] Feigin VL, Norrving B, Mensah GA (2017). Global burden of stroke. Circ Res.

[2] Johnson W, Onuma O, Owolabi M, Sachdev S (2016). Stroke: a global response is needed. Bull World Health Organ.

[3] Boehme AK, Esenwa C, Elkind MS (2017). Stroke risk factors, genetics, and prevention. Circ Res.

[4] Koton S, Tanne D, Bornstein NM, Green MS (2004). Triggering risk factors for ischemic stroke: a case-crossover study. Neurology.

[5] Lerman LO, Chade AR, Sica V, Napoli C (2005). Animal models of hypertension: an overview. J Lab Clin Med.

[6] Nassereddine S, Kassogue Y, Korchi F, Habbal R, Nadifi S (2015). Association of methylenetetrahydrofolate reductase gene (C677T) with the risk of hypertension in Morocco. BMC Res Notes.

[7] Morita H, Taguchi J, Kurihara H (1997). Gene polymorphism of 5, 10-methylenetetrahydrofolate reductase as a coronary risk factor. (Article in Japanese). J Cardiol.

[8] Zaric BL, Obradovic M, Bajic V, Haidara MA, Jovanovic M, Isenovic ER (2019). Homocysteine and hyperhomocysteinaemia. Curr Med Chem.

[9] Ni J, Zhang L, Zhou T, Xu WJ, Xue JL, Cao N, Wang X (2017). Association between the MTHFR C677T polymorphism, blood folate and vitamin B12 deficiency, and elevated serum total homocysteine in healthy individuals in Yunnan Province, China. J Chin Med Assoc.

[10] Panic N, Leoncini E, de Belvis G, Ricciardi W, Boccia S (2013). Evaluation of the endorsement of the preferred reporting items for systematic reviews and meta-analysis (PRISMA) statement on the quality of published systematic review and meta-analyses. PLoS One.

[11] Mejia Mohamed EH, Tan KS, Ali JM, Mohamed Z (2011). TT genotype of the methylenetetrahydrofolate reductase C677T polymorphism is an important determinant for homocysteine levels in multi-ethnic Malaysian ischaemic stroke patients. Ann Acad Med Singap.

[12] Chang G, Kuai Z, Wang J, Wu J, Xu K, Yuan Y, Hu Y (2019). The association of MTHFR C677T variant with increased risk of ischemic stroke in the elderly population: a meta-analysis of observational studies. BMC Geriatr.

[13] L'Abbé KA, Detsky AS, O'Rourke K (1987). Meta-analysis in clinical research. Ann Intern Med.

[14] Casas JP, Bautista LE, Smeeth L, Sharma P, Hingorani AD (2005). Homocysteine and stroke: evidence on a causal link from mendelian randomisation. Lancet.

[15] Kumar A, Kumar P, Prasad M (2015). Association of C677T polymorphism in the methylenetetrahydrofolate reductase gene (MTHFR gene) with ischemic stroke: a meta-analysis. Neurol Res.

[16] Zhang MJ, Li JC, Yin YW (2014). Association of MTHFR C677T polymorphism and risk of cerebrovascular disease in Chinese population: an updated meta-analysis. J Neurol.

[17] Abhinand PA, Manikandan M, Mahalakshmi R, Ragunath PK (2017). Meta-analysis study to evaluate the association of MTHFR C677T polymorphism with risk of ischemic stroke. Bioinformation.

[18] Mattson MP, Shea TB (2003). Folate and homocysteine metabolism in neural plasticity and neurodegenerative disorders. Trends Neurosci.

[19] Wu YL, Hu CY, Lu SS (2014). Association between methylenetetrahydrofolate reductase (MTHFR) C677T/A1298C polymorphisms and essential hypertension: a systematic review and meta-analysis. Metabolism.

[20] Yadav S, Hasan N, Marjot T, Khan MS, Prasad K, Bentley P, Sharma P (2013). Detailed analysis of gene polymorphisms associated with ischemic stroke in South Asians. PLoS One.

[21] Wald DS, Law M, Morris JK (2002). Homocysteine and cardiovascular disease: evidence on causality from a meta-analysis. BMJ.

[22] Trabetti E (2008). Homocysteine, MTHFR gene polymorphisms, and cardio-cerebrovascular risk. J Appl Genet.

[23] Cronin S, Furie KL, Kelly PJ (2005). Dose-related association of MTHFR 677T allele with risk of ischemic stroke: evidence from a cumulative meta-analysis. Stroke.

[24] Casas JP, Hingorani AD, Bautista LE, Sharma P (2004). Meta-analysis of genetic studies in ischemic stroke: thirty-two genes involving approximately 18,000 cases and 58,000 controls. Arch Neurol.

[25] Clarke R, Collins R, Lewington S (2002). Homocysteine and risk of ischemic heart disease and stroke: a meta-analysis. JAMA.

[26] Møller J, Nielsen GM, Tvedegaard KC, Andersen NT, Jørgensen PE (2000). A meta-analysis of cerebrovascular disease and hyperhomocysteinaemia. Scand J Clin Lab Invest.

[27] Xu X, Li J, Sheng W, Liu L (2008). Meta-analysis of genetic studies from journals published in China of ischemic stroke in the Han Chinese population. Cerebrovasc Dis.

[28] Xin XY, Song YY, Ma JF, Fan CN, Ding JQ, Yang GY, Chen SD (2009). Gene polymorphisms and risk of adult early-onset ischemic stroke: a meta-analysis. Thromb Res.

[29] Song Y, Li B, Wang C, Wang P, Gao X, Liu G (2016). Association between 5,10-methylenetetrahydrofolate reductase C677T gene polymorphism and risk of ischemic stroke: a meta-analysis. J Stroke Cerebrovasc Dis.

[30] Li P, Qin C (2014). Methylenetetrahydrofolate reductase (MTHFR) gene polymorphisms and susceptibility to ischemic stroke: a meta-analysis. Gene.

[31] Hassan A, Markus HS (2000). Genetics and ischaemic stroke. Brain.

[32] Morita H, Kurihara H, Tsubaki S (1998). Methylenetetrahydrofolate reductase gene polymorphism and ischemic stroke in Japanese. Arterioscler Thromb Vasc Biol.

[33] Pepe G, Camacho Vanegas O, Giusti B (1998). Heterogeneity in world distribution of the thermolabile C677T mutation in 5,10-methylenetetrahydrofolate reductase. Am J Hum Genet.

[34] Salooja N, Catto A, Carter A, Tudenham EG, Grant PJ (1998). Methylene tetrahydrofolate reductase C677T genotype and stroke. Clin Lab Haematol.

[35] Kostulas K, Crisby M, Huang WX (1998). A methylenetetrahydrofolate reductase gene polymorphism in ischaemic stroke and in carotid artery stenosis. Eur J Clin Invest.

[36] Press RD, Beamer N, Evans A, DeLoughery TG, Coull BM (1999). Role of a common mutation in the homocysteine regulatory enzyme methylenetetrahydrofolate reductase in ischemic stroke. Diagn Mol Pathol.

[37] Lalouschek W, Aull S, Serles W (1999). Genetic and nongenetic factors influencing plasma homocysteine levels in patients with ischemic cerebrovascular disease and in healthy control subjects. J Lab Clin Med.

[38] Harmon DL, Doyle RM, Meleady R (1999). Genetic analysis of the thermolabile variant of 5,10-methylenetetrahydrofolate reductase as a risk factor for ischemic stroke. Arterioscler Thromb Vasc Biol.

[39] Eikelboom JW, Hankey GJ, Anand SS, Lofthouse E, Staples N, Baker RI (2000). Association between high homocyst(e)ine and ischemic stroke due to large- and small-artery disease but not other etiologic subtypes of ischemic stroke. Stroke.

[40] Voetsch B, Damasceno BP, Camargo EC (2000). Inherited thrombophilia as a risk factor for the development of ischemic stroke in young adults. Thromb Haemost.

[41] Zheng YZ, Tong J, Do XP, Pu XQ, Zhou BT (2000). Prevalence of methylenetetrahydrofolate reductase C677T and its association with arterial and venous thrombosis in the Chinese population. Br J Haematol.

[42] Topić E, Simundić AM, Ttefanović M, Demarin V, Vuković V, Lovrencić-Huzjan A, Zuntar I (2001). Polymorphism of apoprotein E (APOE), methylenetetrahydrofolate reductase (MTHFR) and paraoxonase (PON1) genes in patients with cerebrovascular disease. Clin Chem Lab Med.

[43] Zhang G, Dai C (2001). Gene polymorphisms of homocysteine metabolism-related enzymes in Chinese patients with occlusive coronary artery or cerebral vascular diseases. Thromb Res.

[44] Wu Y, Tomon M, Sumino K (2001). Methylenetetrahydrofolate reductase gene polymorphism and ischemic stroke: sex difference in Japanese. Kobe J Med Sci.

[45] Lopaciuk S, Bykowska K, Kwiecinski H (2001). Factor V Leiden, prothrombin gene G20210A variant, and methylenetetrahydrofolate reductase C677T genotype in young adults with ischemic stroke. Clin Appl Thromb Hemost.

[46] Yingdong Z, Zhigang Z, Yang L (2002). Association of plasma homocysteine level and N5,N10-methylenetetrahydrofolate reductase gene polymorphism with cerebral infarction. Chin Med Sci J.

[47] Huang Y, Zhao Yl Yl, Li S (2002). Hyperhomocysteine, methylenetetrahydrofolate reductase gene, and other risk factors in ischemic stroke. (Article in Chinese). Zhonghua Yi Xue Za Zhi.

[48] Grossmann R, Geisen U, Merati G, Müllges W, Schambeck CM, Walter U, Schwender S (2002). Genetic risk factors in young adults with ‘cryptogenic’ ischemic cerebrovascular disease. Blood Coagul Fibrinolysis.

[49] Madonna P, de Stefano V, Coppola A, Cirillo F, Cerbone AM, Orefice G, Di Minno G (2002). Hyperhomocysteinemia and other inherited prothrombotic conditions in young adults with a history of ischemic stroke. Stroke.

[50] McIlroy SP, Dynan KB, Lawson JT, Patterson CC, Passmore AP (2002). Moderately elevated plasma homocysteine, methylenetetrahydrofolate reductase genotype, and risk for stroke, vascular dementia, and Alzheimer disease in Northern Ireland. Stroke.

[51] Szolnoki Z, Somogyvári F, Kondacs A, Szabó M, Fodor L, Bene J, Melegh B (2003). Evaluation of the modifying effects of unfavourable genotypes on classical clinical risk factors for ischaemic stroke. J Neurol Neurosurg Psychiatry.

[52] Li Z, Sun L, Zhang H (2003). Elevated plasma homocysteine was associated with hemorrhagic and ischemic stroke, but methylenetetrahydrofolate reductase gene C677T polymorphism was a risk factor for thrombotic stroke: a multicenter case-control study in China. Stroke.

[53] Choi BO, Kim NK, Kim SH (2003). Homozygous C677T mutation in the MTHFR gene as an independent risk factor for multiple small-artery occlusions. Thromb Res.

[54] Yeh PS, Lin HJ, Li YH, Lin KC, Cheng TJ, Chang CY, Ke DS (2004). Prognosis of young ischemic stroke in Taiwan: impact of prothrombotic genetic polymorphisms. Thromb Haemost.

[55] Wu JM, Wang TG, Li YQ (2004). Genetic mutations of homocysteine metabolism related enzymes in patients with ischemic stroke. (Article in Chinese). Yi Chuan.

[56] Uçar F, Sönmez M, Ovali E, Ozmenoglu M, Karti SS, Yilmaz M, Pakdemir A (2004). MTHFR C677T polymorphism and its relation to ischemic stroke in the Black Sea Turkish population. Am J Hematol.

[57] Baum L, Wong KS, Ng HK (2004). Methylenetetrahydrofolate reductase gene A222V polymorphism and risk of ischemic stroke. Clin Chem Lab Med.

[58] Slooter AJ, Rosendaal FR, Tanis BC, Kemmeren JM, van der Graaf Y, Algra A (2005). Prothrombotic conditions, oral contraceptives, and the risk of ischemic stroke. J Thromb Haemost.

[59] Pezzini A, Grassi M, Del Zotto E (2005). Cumulative effect of predisposing genotypes and their interaction with modifiable factors on the risk of ischemic stroke in young adults. Stroke.

[60] Alluri RV, Mohan V, Komandur S, Chawda K, Chaudhuri JR, Hasan Q (2005). MTHFR C677T gene mutation as a risk factor for arterial stroke: a hospital based study. Eur J Neurol.

[61] Kawamoto R, Kohara K, Oka Y, Tomita H, Tabara Y, Miki T (2005). An association of 5,10-methylenetetrahydrofolate reductase (MTHFR) gene polymorphism and ischemic stroke. J Stroke Cerebrovasc Dis.

[62] Pezzini A, Grassi M, Del Zotto E (2006). Interaction of homocysteine and conventional predisposing factors on risk of ischaemic stroke in young people: consistency in phenotype-disease analysis and genotype-disease analysis. J Neurol Neurosurg Psychiatry.

[63] Sazci A, Ergul E, Tuncer N, Akpinar G, Kara I (2006). Methylenetetrahydrofolate reductase gene polymorphisms are associated with ischemic and hemorrhagic stroke: dual effect of MTHFR polymorphisms C677T and A1298C. Brain Res Bull.

[64] Gao X, Yang H, ZhiPing T (2006). Association studies of genetic polymorphism, environmental factors and their interaction in ischemic stroke. Neurosci Lett.

[65] Hermans MP, Gala JL, Buysschaert M (2006). The MTHFR C677T polymorphism confers a high risk for stroke in both homozygous and heterozygous T allele carriers with type 2 diabetes. Diabet Med.

[66] Panigrahi I, Chatterjee T, Biswas A, Behari M, Choudhry PV, Saxena R (2006). Role of MTHFR C677T polymorphism in ischemic stroke. Neurol India.

[67] Dikmen M, Ozbabalik D, Gunes HV, Degirmenci I, Bal C, Ozdemir G, Basaran A (2006). Acute stroke in relation to homocysteine and methylenetetrahydrofolate reductase gene polymorphisms. Acta Neurol Scand.

[68] Shinjo SK, Oba-Shinjo SM, da Silva R, Barbosa KC, Yamamoto F, Scaff M, Marie SK (2007). Methylenetetrahydrofolate reductase gene polymorphism is not related to the risk of ischemic cerebrovascular disease in a Brazilian population. Clinics (Sao Paulo).

[69] Zhang Y, Xie RP, Shen Y, Fan DS (2008). Interaction between methylenetetrahydrofolate reductase C677T gene polymorphism and sleep duration on risk of stroke pathogenesis. Beijing Da Xue Xue Bao Yi Xue Ban.

[70] Shi C, Kang X, Wang Y, Zhou Y (2008). The coagulation factor V Leiden, MTHFRC677T variant and eNOS 4ab polymorphism in young Chinese population with ischemic stroke. Clin Chim Acta.

[71] Moe KT, Woon FP, De Silva DA (2008). Association of acute ischemic stroke with the MTHFR C677T polymorphism but not with NOS3 gene polymorphisms in a Singapore population. Eur J Neurol.

[72] Biswas A, Ranjan R, Meena A (2009). Homocystine levels, polymorphisms and the risk of ischemic stroke in young Asian Indians. J Stroke Cerebrovasc Dis.

[73] Al-Allawi NA, Avo AS, Jubrael JM (2009). Methylenetetrahydrofolate reductase C677T polymorphism in Iraqi patients with ischemic stroke. Neurol India.

[74] Sabino A, Fernandes AP, Lima LM (2009). Polymorphism in the methylenetetrahydrofolate reductase (C677T) gene and homocysteine levels: a comparison in Brazilian patients with coronary arterial disease, ischemic stroke and peripheral arterial obstructive disease. J Thromb Thrombolysis.

[75] Han IB, Kim OJ, Ahn JY (2010). Association of methylenetetrahydrofolate reductase (MTHFR 677C>T and 1298A>C) polymorphisms and haplotypes with silent brain infarction and homocysteine levels in a Korean population. Yonsei Med J.

[76] Salem-Berrabah OB, Mrissa R, Machghoul S (2010). Hyperhomocysteinemia, C677T MTHFR polymorphism and ischemic stroke in Tunisian patients. Tunis Med.

[77] Isordia-Salas I, Barinagarrementería-Aldatz F, Leaños-Miranda A (2010). The C677T polymorphism of the methylenetetrahydrofolate reductase gene is associated with idiopathic ischemic stroke in the young Mexican-Mestizo population. Cerebrovasc Dis.

[78] Mohamed EHM, Tan KS, Ali JM, Mohamed Z (2011). TT genotype of the methylenetetrahydrofolate reductase C677T polymorphism is an important determinant for homocysteine levels in multi-ethnic Malaysian ischaemic stroke patients. Ann Acad Med Singap.

[79] They-They TP, Nadifi S, Rafai MA, Battas O, Slassi I (2011). Methylenehydrofolate reductase (C677T) polymorphism and large artery ischemic stroke subtypes. Acta Neurol Scand.

[80] Somarajan BI, Kalita J, Mittal B, Misra UK (2011). Evaluation of MTHFR C677T polymorphism in ischemic and hemorrhagic stroke patients. A case-control study in a Northern Indian population. J Neurol Sci.

[81] Arsene D, Găină G, Bălescu C, Ardeleanu C (2011). C677T and A1298C methylenetetrahydropholate reductase (MTHFR) polymorphisms as factors involved in ischemic stroke. Rom J Morphol Embryol.

[82] Xiong L, Hu M, Zhang X (2012). Association between Glu298Asp/677C-T single nucleotide polymorphism in the eNOS/MTHRF gene and blood stasis syndrome of ischemic stroke. Gene.

[83] Aifan L, Hong Z, Yuming X, Xingjuan Z, Xiaoman Z (2012). The association between gene polymorphisms of homocysteine metabolism-related enzymes and ischemic cerebrovascular diseases in Chinese Henan Han population. Life Sci J.

[84] Fekih-Mrissa N, Mrad M, Klai S, Mansour M, Nsiri B, Gritli N, Mrissa R (2013). Methylenetetrahydrofolate reductase (C677T and A1298C) polymorphisms, hyperhomocysteinemia, and ischemic stroke in Tunisian patients. J Stroke Cerebrovasc Dis.

[85] Zhou BS, Bu GY, Li M, Chang BG, Zhou YP (2014). Tagging SNPs in the MTHFR gene and risk of ischemic stroke in a Chinese population. Int J Mol Sci.

[86] Al-Gazally ME, Al-Saadi AH, Radeef AH (2015). Effect of homocysteine on ischemic stroke and myocardial infarction in Iraqi population. Int J Pharmtech Res.

[87] Nissar S, Rasool R, Bashir A, Sameer AS (2015). MTHFR C677T polymorphism and risk of ischemic stroke in Kashmiri population. Hereditary Genet.

[88] Das S, Roy S, Kaul S, Jyothy A, Munshi A (2015). MTHFR gene (C677t) polymorphism in ischemic stroke, its subtypes and hemorrhagic stroke in a South Indian population. Acta Med Int.

[89] Lv QQ, Lu J, Sun H, Zhang JS (2015). Association of methylenetetrahydrofolate reductase (MTHFR) gene polymorphism with ischemic stroke in the Eastern Chinese Han population. Genet Mol Res.

[90] Kumar A, Misra S, Hazarika A (2016). Association between methylenetetrahydrofolate reductase (MTHFR) C677T gene polymorphism and risk of ischemic stroke in North Indian population: a hospital based case-control study. Egypt J Med Hum Genet.

[91] Ma L, Jiang Y, Kong X (2017). Synergistic effect of the MTHFR C677T and EPHX2 G860A polymorphism on the increased risk of ischemic stroke in Chinese type 2 diabetic patients. J Diabetes Res.

[92] Li A, Shi Y, Xu L (2017). A possible synergistic effect of MTHFR C677T polymorphism on homocysteine level variations increased risk for ischemic stroke. Medicine (Baltimore).

[93] Hou J, Zeng X, Xie Y, Wu H, Zhao P (2018). Genetic polymorphisms of methylenetetrahydrofolate reductase C677T and risk of ischemic stroke in a southern Chinese Hakka population. Medicine.

[94] Hashemi SM, Ramroodi N, Amiri Fard H (2019). Common variations in prothrombotic genes and susceptibility to ischemic stroke in young patients: a case-control study in Southeast Iran. Medicina (Kaunas).

[95] Mazdeh M, Khazaie M, Omrani MD (2021). Association between methylene tetrahydrofolate reductase polymorphisms and risk of ischemic stroke. Int J Neurosci.

